# Current Approaches of Pancreatic Cancer Surveillance in High-Risk Individuals

**DOI:** 10.1007/s12029-025-01184-1

**Published:** 2025-02-11

**Authors:** Melissa Heller, Derek A. Mann, Bryson W. Katona

**Affiliations:** 1 Division of Hematology and Oncology, University of Pennsylvania Perelman School of Medicine, Philadelphia, PA USA; 2 Division of Gastroenterology and Hepatology, University of Pennsylvania Perelman School of Medicine, 3400 Civic Center Blvd., 751 South Pavilion, Philadelphia, PA 19104 USA

**Keywords:** Pancreatic cancer, Early detection, Surveillance, High-risk individual

## Abstract

Currently, those recommended to undergo pancreatic cancer (PC) surveillance include appropriately aged individuals at high risk of PC due to an identifiable genetic susceptibility or those without identifiable genetic susceptibility who nonetheless have a strong family history of PC. With increases in identification of individuals at high risk for PC and increased use of PC surveillance in clinical practice, there has been increasing debate about who should undergo surveillance as well as how surveillance should be performed including use of imaging and blood-based testing. Furthermore, there is increasing interest in the outcomes of PC surveillance in high-risk individuals with some studies demonstrating that surveillance leads to downstaging of PC and improvements in survival. In this review, we summarize the current state of PC surveillance in high-risk individuals, providing an overview of the risk factors associated with PC, selection of high-risk individuals for PC surveillance, and the current, but non-uniform, recommendations for performing PC surveillance. Additionally, we review approaches to apply various imaging and blood-based tests to surveillance and the outcomes of PC surveillance.

## Introduction

While pancreatic cancer (PC) is only the tenth most commonly diagnosed cancer, it is the third-leading cause of cancer-related death in the United States [1]. In 2024, it is estimated that 66,440 individuals will be diagnosed with PC in the United States and 51,750 deaths from PC will occur, making up 3.3% of all new cancer diagnoses and 8.5% of all cancer deaths for the year [1], with the vast majority of these PC cases being pancreatic ductal adenocarcinomas (PDAC). Worldwide, there has also been a consistent increase in PC incidence and mortality noted. While longer lifespans have been offered as a possible explanation, especially for the increase in PC incidence in those over the age of 70, this would not explain the increase in PC among younger individuals [2]. Other possible explanations for this increase in PC incidence include increasing rates of obesity, diabetes, and alcohol use [3, 4].

Currently, the 5-year survival rate for PC in the United States is 12.8% [1]. While this survival rate has been increasing, it remains one of the lowest 5-year survival rates among the common cancer types [1]. One of the main reasons this survival rate has been and continues to remain low is that PC is rarely diagnosed at early stages that may permit surgical resection. Additionally, when PC does present with symptoms, these cancers are typically presenting in individuals with late-stage disease. Unfortunately, the majority (> 80%) of new PC diagnoses are made in individuals with unresectable late-stage disease [1].

**Fig. 1:**
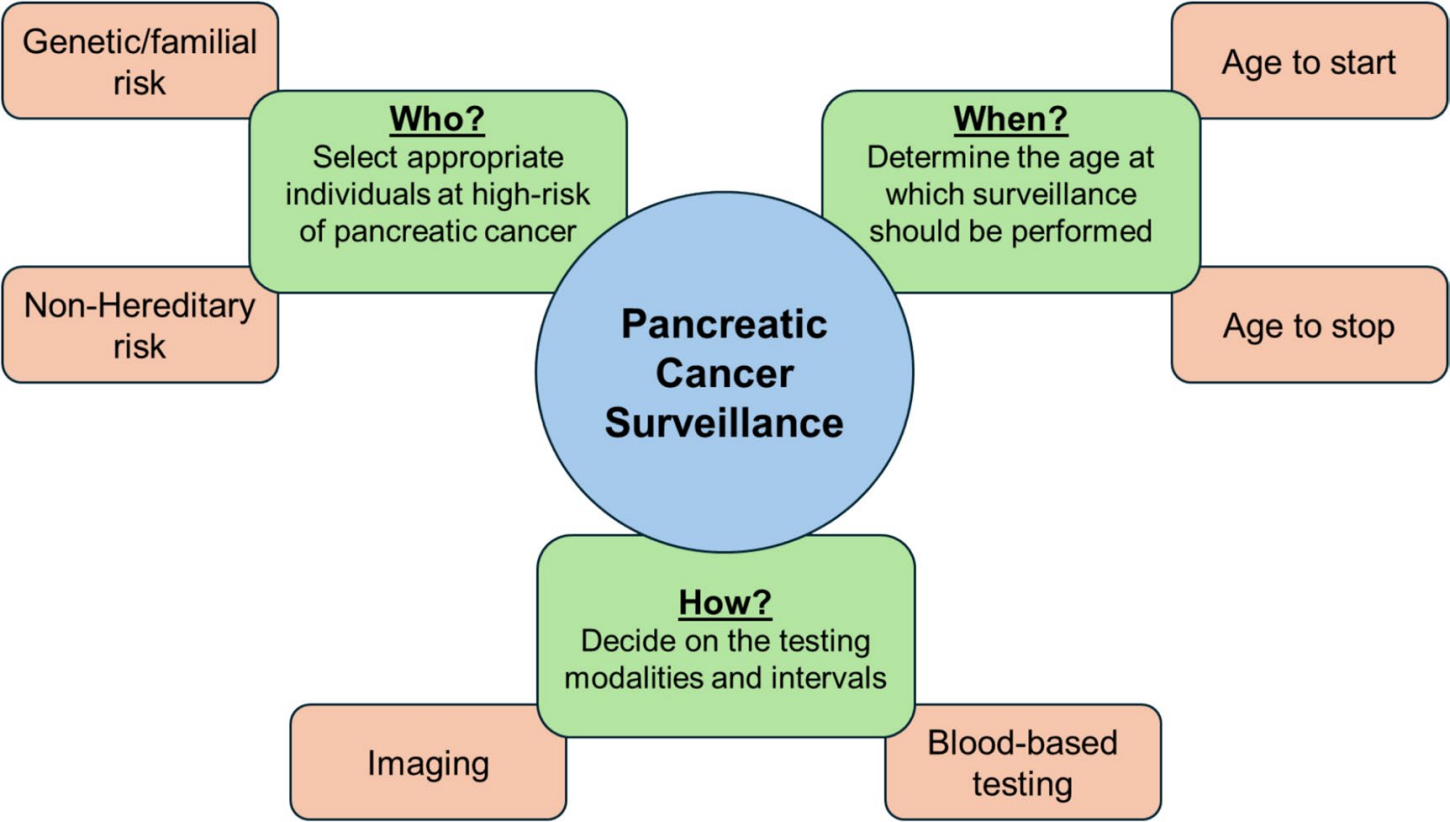
Considerations for high-risk individuals undergoing pancreatic cancer surveillance

PC survival rates increase dramatically when patients present with early-stage or localized disease, with these individuals having a 5-year survival rate of 44% [1]. In fact, 5-year survival for individuals with stage 1A PC is greater than 80% [5]. These dramatic survival differences between early- and late-stage disease demonstrates the dire need for improving access to and implementation of early detection strategies for individuals at increased risk of PC. This also highlights the need to better understand which groups are at the highest risk for PC and might therefore benefit most from PC surveillance. There are many important considerations for high-risk individuals (HRIs) considering PC surveillance including who is eligible for surveillance, when should surveillance be performed, and how should surveillance be performed (Fig. 1). In this review, we will present current data on the known hereditary and environmental risk factors for PC, guidelines on PC cancer surveillance, the current and developing technologies being used for early detection, and the outcomes reported using these strategies and technologies in individuals at high risk for PC.

## Hereditary and Non-Hereditary Risks for Pancreatic Cancer

The first step to determine which individuals are at appropriately high risk to necessitate PC surveillance is to assess the hereditary and non-hereditary risk factors for PC.

### Hereditary Risk for Pancreatic Cancer

It is estimated that 5–10% of PCs are caused by pathogenic germline variants (PGVs) in genes associated with hereditary PC risk. Within cohorts of individuals selected with a history of pancreatic cancer, PGVs were identified in 3–10% [6–8]. This rate of finding PGVs increases to 20% for those who were recommended to pursue germline genetic testing due to a young age at time of diagnosis and/or concerning family history of PC [9]. The genes currently associated with hereditary pancreatic cancer risk are *ATM*, *BRCA1*, *BRCA2*, *CDKN2A*, *MLH1*, *MSH2/EPCAM*, *MSH6*, *PMS2*, *STK11*, *PRSS1*, and *TP53* [6–9]. Current lifetime risks for PC associated with PGVs in these genes are available via the National Comprehensive Cancer Network (NCCN) guidelines and are summarized in Table 1. For individuals with a diagnosis of PC, determining if there is a PGV in a PC risk gene is essential for identifying a contributing factor to explain why the PC developed, allowing medical management recommendations to be made for additional cancer risks associated with the gene, and understanding risk for blood relatives who may have also inherited the PGV. Additionally, identifying PGVs may have treatment implications, such as the use of poly (ADP-ribose) polymerase inhibitors (PARPi), which are associated with a significant increase in progression-free survival in *BRCA1* and *BRCA2* PGV carriers [10, 11].

**Table 1:** Lifetime risk of PC among hereditary cancer syndromes

Gene	Lifetime risk of PC [12]
*ATM*	~ 5–10%
*BRCA1*	$\le$ 5%
*BRCA2*	5–10%
*CDKN2A*	> 15%
*MLH1*	< 5–10%
*MSH2/EPCAM*	< 5–10%
*MSH6*	< 5–10%
*PMS2*	Insufficient Evidence
*PALB2*	2–5%
*STK11*	> 15%
*TP53*	~ 5%

#### *BRCA1*, *BRCA2*, and *PALB2*

PGVs in *BRCA1* and *BRCA2* disrupt homologous recombination-mediated DNA repair and cause hereditary breast and ovarian cancer syndrome (HBOC). The prevalence of PGVs in *BRCA1* and *BRCA2* range from 1 in 200 to 1 in 400 within different populations [13]. For individuals with Ashkenazi Jewish ancestry, where there are three known founder PGVs within these genes [*BRCA1* 185delAG (c.68_69delAG), *BRCA1* 5382insC (c.5266dupC), and *BRCA2 *6174delT (c.5946delT)], the risk of carrying a PGV may be as high as 1 in 44 [13]. Individuals with PGVs in *BRCA1* or *BRCA2* have a well-established increased risk of breast, ovarian, prostate, and pancreatic cancers. For PC risk, *BRCA2* confers a higher risk with an approximate 3.3- to 8.9-fold increased risk compared to the general population and lifetime risk of up to 5–10% [12, 14, 15]. *BRCA1*, in comparison, confers a lower risk with an approximate 2.1-fold increased risk and a lifetime risk of up to 5% [12, 14]. More recent data has demonstrated that the cumulative PC incidence in women between age 40 and 80 is 2.2% and 2.7% for *BRCA1* and *BRCA2* carriers, respectively [16]. Another gene associated with breast, ovarian, and pancreatic cancers that is also involved in the homologous recombination DNA repair pathway is *PALB2.* PGVs in the *PALB2* gene are associated with a 2.4-fold increased risk for PC or an approximate 2–5% lifetime risk [12, 17].

#### Lynch Syndrome

Lynch syndrome (LS) is the most common cause of hereditary colorectal and uterine cancers, with approximately 1 in 270 individuals being affected [18]. Individuals with a diagnosis of LS have a PGV in either the *MLH1*, *MHS2*, *MSH6*, *PMS2*, or *EPCAM* genes. While colorectal and uterine are the most common cancers observed in LS, individuals with LS may be at increased risk of other cancers including pancreatic, ovarian, gastric, urothelial, brain, and sebaceous skin cancer among others. There are also genotype-specific cancer risks in LS with PGVs in the *MLH1*, *MSH2*, or *EPCAM* genes being associated with the highest risk for LS-associated cancers, while those in *MSH6* and *PMS2* are associated with a more moderately increased risk [18, 19]. Data from the Prospective Lynch Syndrome Database indicated that *MLH1* PGV carriers have a lifetime risk of PC estimated at 6.2%, with *MSH2*, *EPCAM*, *MSH6*, and *PMS2* all having an estimated lifetime risk up to 1.6%, similar to the general population [19, 20]. However, other research highlights that *MLH1*, *MSH2*, *EPCAM*, and *MSH6* are all associated with a lifetime risk of PC of up to 5–10%, with no increased risk observed for *PMS2* carriers compared to the general population [12].

#### *ATM*

Heterozygous *ATM* PGV carriers have a moderately increased risk for breast cancer as well as an approximate ~ 5–10% lifetime risk for PC [12, 21]. Individuals who are homozygous carriers of a PGV in the *ATM* gene have a diagnosis of ataxia telangiectasia, a childhood onset disorder associated with ataxia, lowered immunity, and an increased risk for cancers [12].

#### Peutz-Jeghers Syndrome

Peutz-Jeghers syndrome (PJS) is caused by a PGV in the *STK11* gene. PJS is characterized by mucosal hyperpigmentation and hamartomatous gastrointestinal polyposis including small bowel polyps that can lead to intussusception. Individuals with a diagnosis of PJS are at an increased risk of breast, pancreatic, gastric, small bowel, and colorectal cancer among others [22]. Regarding PC specifically, PJS is associated with a 132-fold increased risk for PC with up to an approximate 36% lifetime risk, making it one of the highest risk PC predisposition syndromes [23, 24].

#### *CDKN2A*

*CDKN2A* is associated with the familial atypical multiple mole melanoma (FAMMM) syndrome. *CDKN2A* encodes two proteins, p16(INK) and p14(ARF), both of which are tumor suppressors [25]. PGVs within *CDKN2A* can affect p16, p14, or both p16 and p14 protein functions depending on where in the gene the variant is located. PGVs in *CDKN2A* that affect only the tumor-suppressor protein p16(INK) confer increased risk for melanoma and PC. PGVs affecting only the tumor-suppressor protein p14(ARF) are associated with nerve sheath tumors, while those affecting both p16 and p14 are associated with pancreatic cancer, atypical nevi, melanoma, brain tumors, gallbladder tumors, thyroid cancer, and sarcomatous nerve sheath tumors [25]. PC risk for *CDKN2A* PGVs affecting p16 or p16/p14 is increased 13- to 22-fold with a lifetime risk of up to approximately 17%, making these individuals, similar to those with PJS, one of the groups with the highest risk of PC [26].

#### Hereditary Pancreatitis

Hereditary pancreatitis (HP) is typically characterized by recurrent episodes of acute pancreatitis that can start in childhood, leading eventually to chronic pancreatitis. *PRSS1* is the most common gene associated with HP, identified in up to 80% of those affected worldwide [27]. Lifetime risk for PC is increased for *PRSS1* PGV carriers who display a pancreatitis phenotype, with lifetime risk for PC estimated to be 44–52% [28, 29]. PGVs in the *SPINK1* gene have also been associated with an increased risk in pancreatic cancer, although controversy exists over *SPINK1* being the sole driver of this risk [27]. Overall, the lifetime PC risk of PGVs in other HP-associated genes such as *CFTR*, *CRTC*, *CPA1*, and *CASR* remains uncertain.

#### Li-Fraumeni Syndrome

Li-Fraumeni syndrome (LFS) is caused by PGVs in the *TP53* gene and is one of the highest risk cancer predisposition syndromes. LFS has multiple known cancer risks including sarcoma, breast, central nervous system tumors, and adrenocortical tumors [12]. Individuals with LFS may develop cancers at young ages and may develop multiple cancers over their lifetimes. While PC is not one of the classic cancers associated with LFS, the lifetime risk is estimated to be at least 5% for those with LFS [12].

#### SNPs

While all of the genes discussed so far are associated with increased PC risk, it is also possible that other less penetrant genetic changes may in aggregate also contribute to increased PC risk. As such, single nucleotide polymorphisms (SNPs) may give further insight into PC risk, especially when utilized in the context of polygenic risk scores (PRS). Use of PRS to assist in elucidating those at increased risks of PC without an identifiable monogenic variant may in the future be important for personalizing PC surveillance, especially with the emerging evidence of additional susceptibility loci [30].

#### Familial Pancreatic Cancer

Familial pancreatic cancer (FPC) is defined as a family with two or more individuals with PC who are first-degree relatives without a PGV identified in a PC susceptibility gene. For an individual to have this diagnosis, the individual themselves must have a diagnosis of PC, or be a first-degree relative to at least one of the affected individuals [31–33]. Estimating risk for unaffected individuals meeting FPC criteria depends on the number of first-degree relatives diagnosed with PC. While individuals with one first-degree relative diagnosed with PC have a 4.5-fold increased risk of developing PC, those with two or three first degree relatives diagnosed with PC have a lifetime risk that is increased by 6.4-fold and 34-fold, respectively [31–33].

### Non-Hereditary Risk Factors for Pancreatic Cancer

Beyond PGVs in known hereditary cancer syndromes associated with increased PC risk, multiple environmental factors may also confer an increased risk.

#### Pancreatitis

Chronic pancreatitis is characterized by chronic inflammation of the pancreatic parenchyma. This inflammation increases the risk for the development of somatic mutations such as in *KRAS* and the development of pancreatic intraepithelial neoplasm and promotes acinar-to-duct metaplasia, all of which promote PC development [28]. A history of chronic pancreatitis is associated with a 2.7–13.3-fold increased risk of PC, with higher risks associated with concurrent factors such as smoking, excess alcohol consumption, and late-onset diabetes mellitus [28].

#### Smoking

Regular cigarette or pipe tobacco use is known to increase risk for PC, with cigarette use being associated with the highest risk [34]. Individuals with a current history of smoking are at a 1.66-fold increased risk for PC and former smokers at 1.4-fold increased risk, compared to those who never smoked [35]. For those with a current smoking history, the increased PC risk is proportional to the number of cigarettes smoked per day, with those over 35 cigarettes per day having a 3.0-fold increase risk [34, 36]. For those who stopped tobacco use, the risk for PC decreases as time from smoking cessation increases, with those 10–20 years since smoking cessation having a similar risk to never smokers [34]. Overall, a history of smoking is thought to be associated with 11–32% of PC cases [37].

#### Alcohol

Individuals with a personal history of heavy alcohol use (3 or more drinks per day) were found to be at a 1.29-fold increased risk of PC, and those with light alcohol use (less than 1 drink per day) were found to have a risk similar to non-drinkers [35]. The Pancreatic Cancer Case Control Consortium (PanC4) found a 1.6-fold increased risk for those consuming nine or more alcoholic beverages per day compared to one per day [38].

##### Weight

Increased weight and body mass index (BMI) throughout life has been associated with an increased risk of PC. A Danish study revealed an increased risk for PC for those with high childhood BMI while an American Association of Retired Persons (AARP) study showed an increase risk for PC in those who gain weight over age 50 [39, 40]. Additionally, the Nurse’s Health study revealed that individuals with a BMI of ≥ 30 kg/m^2^ had a 1.72-fold increased risk for PC compared to those with a BMI of < 23.0 kg/m^2^[41]. Within one diverse study population, an increased risk for PC was seen for those who reported an increase in weight/BMI during adulthood in certain races/ethnicities, namely, Japanese Americans and Latinos, while for African American/Black, White, and Hawaiian patients, this difference was not significant [42].

##### Diabetes

For individuals with long-standing type 2 diabetes mellitus (DM), the risk for PC is roughly 2-fold higher than the general population [43–45]. However, the risk is significantly higher for those within 3 years of new-onset diabetes mellitus (NOD) [4, 45]. Although determining the exact start date of type 2 DM can be difficult as many individuals may have a preceding asymptomatic period where they remained undiagnosed, it is estimated that 1% of those with NOD occurring after the age of 50 will have a diagnosis of PC within 3 years [46]. When comparing patients with a history of PC to controls, the overall prevalence of type 2 DM was much higher (47% vs 7%) for PC patients. Furthermore for those with a history of type 2 DM, NOD (within 2 years for this study) was more commonly seen in the PC population compared to controls (74% vs 53%) [47]. In an Israeli study of 2.3 million individuals, the risk of PC within the first year of type 2 DM diagnosis was very high, with men and women having a roughly 15-fold and 14-fold increased risk, respectively [48]. Other meta-analyses have identified similar findings, with roughly five- to seven-fold increased risk being reported in the first year after type 2 DM diagnosis [45, 49, 50].

As most of the research of DM-associated PC risk was completed in predominately White populations, a more diverse study that looked at a more heterogenous population from 2006 to 2016 also identified a sevenfold increased risk for PC in newly diagnosed type 2 DM patients [51]. This study also stated that those who developed PC had a more rapid increase in glucose levels and HbA1c in the month before PC diagnosis [51]. When looking specifically at African American/Black and Latino populations, increased risks for PC of 4-fold and 3.5-fold after a recent diagnosis of type 2 DM, respectively, were reported, which was 2.3-fold higher than those with long-standing type 2 DM [52].

## Who Should Undergo Pancreatic Cancer Surveillance

Selecting the appropriate individuals for surveillance who are at increased risk of PC is incredibly important, and historically, individuals at a 10-fold increased risk of developing PC were considered eligible for PC surveillance [53, 54]. However, more recent guidelines including the International Cancer of the Pancreas Screening (CAPS) Consortium have advocated that those with a 5-fold increase in risk, equating to a lifetime risk of approximately 5% or higher, should consider pancreatic surveillance if they are of an appropriate age [55]. At this time, PC surveillance is offered to those with genetic and/or familial risk of developing PC rather than those at average risk or with clinical risk factors alone [56]. In fact, the US Preventative Services Task Force has specifically recommended against PC screening in the asymptomatic general population [57].

There are multiple professional guidelines that suggest who, when, and how to surveil for PC in high-risk individuals (Table 2), and there are important differences to consider between these guidelines. The recommendations per guideline summarized in this review focus on those with identifiable genetic risk for PC or FPC with unidentifiable genetic risk.

**Table 2:** Summary of pancreatic cancer surveillance recommendations

Gene		NCCN	CAPS	AGA	ASGE
*ATM*	Age to start	50	45–50	50	50
	FHx required	No	FDR	FDR	FDR or SDR
*BRCA1*	Age to start	50	45–50	50	50
	FHx required	FDR or SDR	FDR	FDR	No
*BRCA2*	Age to start	50	45–50	50	50
	FHx required	No	FDR	FDR	No
*PALB2*	Age to start	50	45–50	50	50
	FHx required	FDR or SDR	FDR	FDR	No
*STK11*	Age to start	30–35	40	30–35	30–35
	FHx required	No			
*CDKN2A*	Age to start	40			
	FHx required	No			
*MLH1*	Age to start	50			
	FHx required	FDR or SDR	FDR	FDR	FDR or SDR
*MSH2*	Age to start	50			
	FHx required	FDR or SDR	FDR	FDR	FDR or SDR
*MSH6*	Age to start	50			
	FHx required	FDR or SDR	FDR	FDR	FDR or SDR
*EPCAM*	Age to start	50	–	–	50
	FHx required	FDR or SDR	–	–	FDR or SDR
*PMS2*	Age to start	–	50	–	
	FHx required	–	FDR	–	FDR or SDR
*TP53*	Age to start	50	–		
	FHx required	FDR or SDR			
*PRSS1*^a^	Age to start	40 (or 20 years after onset of first episode of pancreatitis)			
	FHx required	No			
Familial pancreatic cancer	Age to start	–	50 or 55	50	50
	FHx required	–	1 FDR who has a FDR with PC	1 FDR who has ≥ 2 affected relatives	≥ 1 FDR

FHx required refers to if each guideline specifies needing a family history of PC in order to qualify for surveillance. For all ages other than for PRSS1, the age to start surveillance is the age listed or 10 years prior to the youngest relative diagnosed with PC

^a^Only with a clinical phenotype consistent with hereditary pancreatitis [12, 19, 55, 58–60]

The National Comprehensive Cancer Network (NCCN), the International Cancer of the Pancreas Screening (CAPS) consortium, the American Gastroenterological Association (AGA), and the American Society for Gastrointestinal Endoscopy (ASGE) have all put out recent guidelines and recommendations for who should undergo PC surveillance. For those with PJS, caused by a PGV in *STK11*, NCCN, ASGE, and AGA recommend beginning surveillance at age 30–35, while CAPS recommends initiating at age 40. No family history of PC is required by any guideline society for PC surveillance in PJS. For individuals with a PGV in *CDKN2A*, all societies agree that surveillance starts at age 40 and that a family history of PC is not required [12, 58–60].

For individuals with *ATM*, *BRCA1*, *BRCA2*, and *PALB2* PGVs, there is relative agreement between the guidelines that if PC surveillance is performed, the age of initiation should be between 45 and 50 years old with most guidelines favoring starting at age 50, or 10 years younger than the youngest PC in the family. However, there are more substantial differences among the guidelines pertaining to whether a family history of PC should be required, and what the extent of that family history should be [12, 58–60]. NCCN recently modified their guidelines in 2024 so that a family history of PC is no longer required for those with an *ATM* or *BRCA2* PGV, though NCCN does note that a first or second degree relative (from the same side of the family as the PGV) must be affected with PC in order for those with a *BRCA1* or *PALB2* PGV to qualify for surveillance [12]. ASGE, on the other hand, does not require family history for *BRCA1*, *BRCA2*, and *PALB2* carriers, but does recommend that *ATM* PGV carriers only consider surveillance if there is a first or second degree relative with PC [59].

Recommendations for PC surveillance across guidelines for individuals with LS caused by PGVs in *MLH1*, *MSH2/EPCAM*, and *MSH6* are relatively consistent, with all guidelines recommending surveillance begin at age 50 and that there be a family history of PC*.* With respect to family history, NCCN and ASGE require a first or second degree relative with PC, while AGA and CAPS require a first degree relative. Recommendations for *PMS2* are less consistent, with NCCN and AGA not recommending any surveillance be performed at all, and CAPS and ASGE recommending surveillance begin at age 50. CAPS also requires a first degree relative for *PMS2* carriers, and ASGE requires a first or second degree relative [19, 58–60].

Regarding LFS, caused by a PGV in *TP53,* NCCN is the only society to officially recommend surveillance, recommending starting PC surveillance at age 50 in those with a family history of PC in either a first or second degree relative [12]. In the setting of hereditary pancreatitis with a clinical pancreatitis phenotype, if this is caused by a PGV in *PRSS1,* all societies recommend surveillance regardless of family history beginning at age 40, or 20 years after the onset of the first episode of pancreatitis. This guidance does not apply to those who have a PGV in *PRSS1* but do not have a clinical phenotype of pancreatitis [12, 58–60].

Surveillance criteria for those who meet FPC criteria differs among the guidelines in both age of initiation and the definition of what criteria defines families with FPC. NCCN notes emerging data supporting surveillance for those with FPC, though does not explicitly recommend consideration of surveillance in this population [12]. CAPS recommends initiation of surveillance at age 50–55 for individuals with one first degree relative who also has a first degree relative with PC (in the same lineage) [60]. AGA recommends surveillance begin at age 50 for those who have one first degree relative among at least two relatives with PC in the same lineage [58]. ASGE also recommends surveillance begin at age 50 for FPC families where the individual being considered for surveillance should be an FDR of at least one of a pair of FDRs with PC in the family [59]. The NCCN and AGA further note that surveillance should be performed at an experienced, high-volume center only after an in-depth discussion of limitations, risks, and benefits [12, 58].

While not summarized in Table 2, the European Society for Medical Oncology (ESMO) also recommends high-risk individuals consider pancreatic cancer surveillance with EUS and/or MRI, typically beginning at age 50 or 10 years prior to the earliest diagnosis in the family [61]. This is due to continual demonstration that surveillance allows for higher rates of surgical resection and early data suggesting longer term survival [57, 59, 62–64]. ESMO is in agreement with NCCN and AGA with recommending surveillance to be performed at an experienced, high-volume center [61].

## How Should Surveillance Be Performed

Current guidelines recommend that PC surveillance should be performed with regular imaging of the pancreas; however, there has also been increasing research into the use of non-invasive blood-based tests for PC surveillance. The most common modalities for imaging the pancreas include endoscopic ultrasonography (EUS), MRI abdomen with IV contrast and MRCP (herein referred to as MRI), and pancreatic protocol CT (herein referred to as CT), with each of these techniques being limited in their scope of diagnosis and staging [65]. Current guidelines recommend that surveillance is performed with EUS and/or MRI as the primary imaging modalities, typically on an annual basis [58, 60]. There are, however, limited guidance on specific protocols for how these imaging modalities should be used or reported. Recently, the Pancreatic Cancer Early Detection (PRECEDE) consortium published recommendations for a standardized protocol and reporting template for both MRI and EUS [66]. Additionally, there are multiple longitudinal studies capturing outcomes of surveillance, which high-risk individuals (HRIs) should be encouraged to enroll in if feasible, and which will be critical to ensuring future advancement of the field (Table 3).

**Table 3:** Select current pancreatic cancer early detection trials in North America for high-risk individuals

Pancreatic cancer trial	Study site	Study population	ClinicalTrials.gov identifier
CAPS5: The Cancer of the Pancreas Screening-5 Study [64, 67]	Multisite	HP, PJS, FPC; *ATM*, *BRCA1*, *BRCA2*, *CDKN2A*, *EPCAM*, *MLH1*, *MSH2*, *MSH6*, *PALB2*, *PMS2*, *PRSS1/2*	NCT02000089
Preliminary Evaluation of Screening for Pancreatic Cancer in Patients with Inherited Genetic Risk [68]	Single site	*ATM*, *BRCA1*, *BRCA2*, *PALB2*	NCT02478892
A Pancreatic Cancer Screening Study in Hereditary High Risk Individuals [69]	Single site	FPC; *ATM*, *BRCA1*, *BRCA2*, *CDKN2A*, *MLH1*, *MSH2*, *MSH6*, *PALB2*, *PMS2*, or similar high risk gene mutation with ≥ 1 FDR or SDR with PC	NCT03250078
PRECEDE: Pancreatic Cancer Early Detection Consortium [70]be	Multisite	FPC; *ATM*, *BRCA1*, *BRCA2*, *EPCAM*, *MLH1*, *MSH2*, *MSH6*, *PALB2*, *PMS2* ± FDR/SDR with PC; FAMMM (*CDKN2A*); PJS (*STK11*); HP (*PRSS1*)	NCT04970056
Pancreas Scan: Pancreatic Cancer Screening for At-Risk Individuals (PancreasScan) [71]	Single site	FPC; PJS, FAMMM; HBOC (clinical criteria or *BRCA1*, *BRCA2*, *PALB2*) + FDR/SDR; LS or *ATM* mutation + FDR; HP	NCT05006131
Pilot Study of Pancreatic Cancer Screening [72]	Single site	*ATM*, *BRCA1*, *BRCA2*, *PALB2* ± a family history of PC	NCT05058846
Prospective Screening for Pancreatic Ductal Adenocarcinoma in High- Risk Individuals [73]	Single site	*ATM*, *BRCA1*, *BRCA2*, *EPCAM*, *MLH1*, *MSH2*, *MSH6*, *PMS2*, *TP53 (*+ *FDR/SDR); CDKN2A; STK11; FPC (*≥ *2 FDR*, *1 FDR* + *1 SDR,* ≥ *3 FDR/SDR); PRSS1 and HP*	NCT06122896

### Comparing EUS and MRI

One of the most common areas of debate arises when deciding on which imaging test to use for PC surveillance, and whether EUS or MRI is the preferred imaging modality. As current guidelines do not specify using one modality over the other, it is important to have an informed discussion about the two modalities with individuals undergoing surveillance to help determine which imaging modality is most appropriate for a specific individual.

EUS is a highly sensitive test that is helpful for oncologic staging as it is excellent at determining tumor size and extent of lymph node and vascular involvement [65, 74]. Advantages of EUS include the ability to perform concurrent fine needle aspiration or biopsy (FNA or FNB) of an observed lesion, and there is also some evidence that EUS may be better at detecting solid lesions of the pancreas compared to MRI [74]. Additionally, many individuals undergoing PC surveillance due to genetic risk, including those with LS, PJS, and LFS, may need regular upper endoscopic surveillance separate from PC surveillance, and therefore, EUS can easily be performed concurrently with a surveillance upper endoscopy [12, 75]. Disadvantages of EUS include that it is an invasive procedure with more risk than an MRI and it requires sedation. Use of sedation will also mandate that the individual undergoing EUS has an escort home, which may provide a barrier to successfully completing surveillance. Additionally, there may be situations where EUS would not be able to visualize the entire pancreas, especially in the presence of foregut surgery such as with a total gastrectomy or Roux-en-Y gastric bypass. EUS also relies on provider training and expertise and shows interobserver variability, even among experienced providers [76].

MRI is another surveillance modality to consider for HRIs [77]. MRI image resolution quality has improved over time without a significant increase in artifacts [78, 79]. Advantages of using MRI include being logistically easier, as the patient does not need sedation, it is a non-invasive test, and it may be able to be performed along with other abdominal surveillance imaging. One systematic review and meta-analysis established MRI to be superior in terms of sensitivity, specificity, and diagnostic accuracy as compared to EUS and CT [80]. However, MRIs also have their challenges due to the small size of the pancreas, which may lead to suboptimal visualization of parts of the pancreas on MRI imaging [77]. Additionally, early-stage PCs may be challenging to see on imaging as they can appear as very subtle abnormalities. Interpretation of MRIs can also be reader-dependent, and it is recommended that radiologists take this into account when reading a MRI that is performed as a surveillance exam. Likely with the increase in surveillance programs, radiologists will become more familiar with imaging features of early pancreatic cancer on MRI and will be able to better detect these features [81]. Another obvious limitation with MRI is the concern for false positives due to benign lesions, and the management of these incidentally identified findings. MRIs do outperform EUS in identification of pancreatic cysts; however, this is largely driven by identification of small sub-centimeter pancreatic cysts which have limited clinical significance [74]. The most common cysts identified in the pancreas are intraductal papillary mucinous neoplasms (IPMNs). While a small subset of IPMNs may progress from low-grade to high-grade dysplasia, then to invasive adenocarcinoma, most IPMNs remain unchanged overtime [82]. There have since been guidelines created with recommendations for how to manage these lesions, concerning characteristics to assess for and specific radiologic features to comment on in MRI reports [83]. MRIs also do not visualize the upper GI tract, meaning that for HRIs who need upper GI surveillance, additional testing would need to be performed with an upper endoscopy [75].

### Blood Tests

#### Hemoglobin A1C/Blood Glucose

Though the association between diabetes mellitus (DM) and PC susceptibility has been recognized for multiple years, not all individuals with PC have concurrent DM, and only a minority of individuals with DM develop PC. Nonetheless, it is well established that DM is a risk factor for PC, and this association can go both ways with some PCs causing a physiologically distinct PC-related DM [84, 85]. In fact, more recent data has demonstrated that a new onset of type 2 DM may precede the diagnosis of PC by up to 3 years; furthermore, supporting the correlation is that new-onset type 2 DM may resolve after a PC resection [52, 86]. There is also evidence that HgbA1c levels taken at presentation from patients in a multidisciplinary pancreas clinic were significantly higher in those with PC compared to those without PC. Additionally, those with PC are more likely to present with a mean HgbA1c in the pre-diabetic range (5.7–6.4%) [87].

Given the associations between DM/pre-DM and PC, there has been increasing use of fasting glucose and/or HgbA1c monitoring among high-risk individuals undergoing PC surveillance, with monitoring typically performed on at least an annual basis. However, at this time, fasting glucose and/or HgbA1c monitoring is not formally recommended in PC surveillance guidelines. One recent study from the Netherlands showed that longitudinal monitoring of glucose levels provided no added value to HRIs undergoing PC surveillance; however, additional studies in larger and more diverse populations are needed [88]. Furthermore, whether use of DM/pre-DM screening will improve PC early detection remains uncertain and needs evaluation in prospective longitudinal studies. Additionally, the field needs guidance for how alterations in fasting glucose and/or HgbA1c should be interpreted in HRIs and how these changes should impact future surveillance.

#### CA19-9

Carbohydrate antigen 19–9 (CA19-9) is a biomarker for a number of gastrointestinal cancers and is the most commonly followed biomarker for individuals with a diagnosis of PC [89]. In those with PC, CA19-9 has been shown to correlate with the cancer stage and tumor size, allowing the ability to use CA19-9 in conjunction with other clinical features and imaging to assess responses and prognosis [90, 91]. Typically, a concentration of CA19-9 above 500 U/mL results from a neoplasm, though this marker cannot solely be used to prove the presence or absence of cancer due to insufficient sensitivity and specificity [91]. However, elevated CA19-9 can also result from a number of benign conditions, such as pancreatitis, pancreatic cysts, DM, liver disease, benign cholestatic disease, and other gynecologic, pulmonary and urologic diseases [92–94]. Several non-pancreatic malignant processes can also cause elevated CA19-9, such as salivary gland, thyroid, pulmonary, gynecological, urological, gastrointestinal, and hepatocellular cancers [94–96].

There are some concerns with using CA19-9 as a screening test due to elevations being caused by benign etiologies or individuals having elevated CA19-9 at baseline. Because of this, it can be challenging to establish whether an elevation in CA19-9 is due to a non-malignant disease process or an underlying PC [92]. Further, a subgroup of up to 20% of individuals will not express CA19-9 due to being Lewis antigen null; thus, CA19-9 would not be a useful longitudinal marker in this cohort [92, 97]. While CA19-9 may be an anchor marker for pancreatic cancer, it may be a more useful test when used in conjunction with imaging such as EUS or MRI/MRCP, and thus, further study is needed to determine its role in a PC surveillance protocol [91, 98].

Recent work has explored utilizing SNPs in fucosyltransferase (FUT) enzyme groups to personalize an HRI’s CA19-9 reference range [99]. Specifically, individuals can be divided into three genetic subgroups of CA19–9 levels: *FUT3-null* (where CA19-9 is not expressed), *FUT2-null* (where individuals may have elevated CA19-9 levels at baseline), and those with intact *FUT2* and *FUT3* [100–102]. By incorporating an individual’s FUT group along with their CA19-9 levels, the sensitivity of detecting PC does increase, though the specificity is slightly reduced [103]. Use of *FUT2*/*FUT3* genotyping in the prospective CAPS5 trial is currently ongoing (Table 3).

#### DUPAN-2

As up to 20% of individuals do not express CA19-9 due to being Lewis antigen null, another marker that may have utility in place of CA19-9 is DUPAN-2. DUPAN-2 is a precursor of CA19-9, and while it is not regularly used in the United States, DUPAN-2 is utilized in Japan as a marker of PC burden in those not expressing CA19-9. Ando et al. recently studied the utility of utilizing DUPAN-2 levels according to an individual’s *FUT2* or *FUT3* variant status [104]. The authors found that combining CA19-9 with DUPAN-2 and classifying subjects according to their FUT group yielded 81.3% and 75.2% sensitivities for detection of stage I and stage II PC and 98.0% and 97.4% specificity, which was favorable compared to CA19-9 alone [104]. When looking at diagnostic performance only in stage 1 PC, the sensitivity of DUPAN-2 was superior when using the functional FUT group cutoffs compared to the uniform cutoffs. Overall, among all stage 1 cases, the combined FUT/CA19-9/DUPAN-2 test had a sensitivity of 62.0% vs 49.3% for the CA19-9/DUPAN-2 group without the FUT gene test [104]. While the authors suggest that the high diagnostic accuracy of the combined FUT/CA19-9/DUPAN-2 test approaches the diagnostic performance required for further evaluation in a clinical trial, it will likely be most useful when combined with pancreatic imaging for surveillance purposes [104].

#### IMMray PanCan-d

The first commercial blood test specifically designed for PC early detection was the IMMray PanCan-d test, combining an 8-plex biomarker signature with CA19-9. Possible results from this test included a positive, negative, borderline, or test not performed result. Initial studies using this test showed a 99% specificity and 92% sensitivity for PC diagnosis [105]. Overall, the negative predictive value (NPV) of IMMray remained > 99% regardless of estimated disease prevalence (1% vs 2%), and with borderline results considered as either a negative result or a positive result. The positive predictive value (PPV) ranged from 35.3 to 52.4% when borderline results were not considered positive results and 6.4 to 12.1% when borderline results were considered positive results [97]. While the NPV was robust and ideal for a blood-based test, the PPV was sub-optimal, especially when borderline results were considered positive results. At present, the IMMray PanCan-d test is no longer commercially available.

#### ctDNA

ctDNA is currently used to monitor tumor burden in patients affected with multiple types of cancer, and its potential to also serve as an early detection tool is currently under investigation in numerous studies including investigating ctDNA as a single-cancer early detection (SCED) and/or multi-cancer early detection test (MCED). Advantages of using blood-based SCEDs/MCEDs include that they are non-invasive and MCEDs have the potential to detect multiple different tumor types, though there are concerns about costs, insurance coverage of the SCEDs/MCEDs and subsequent work-up of any positive results, and recommended frequency of performing these tests. However, although there are multiple SCEDs/MCEDs on the market or coming to market, at this time, none of these tests are currently recommended for HRIs undergoing PC surveillance [106].

Two of the most well-studied ctDNA-based MCED tests are CancerSEEK and Galleri [107, 108]. CancerSEEK is a blood-based multi-analyte test simultaneously evaluating both the levels of eight proteins and the presence of pathogenic variants in cancer driver genes in ctDNA. As for PC, CancerSEEK was used to study over 1000 patients with a variety of cancer types (stage I–III) prior to any treatment, with 93 patients having a PC diagnosis (4 stage I, 83 stage II, and 6 stage III) [109]. PCs were detected with approximately 70% sensitivity on CancerSEEK, which is higher than the test’s average for all cancer types (55%).

The Pathfinder study utilizing the Galleri test studied more than 6000 subjects and showed a false-positive rate of less than 1% (1.4% of all subjects had a positive test with 38% of these individuals having a cancer diagnosis). Approximately 50% of the subjects with any cancer diagnosis were stage I or stage II at diagnosis [108]. In this study, there was only one PC diagnosed (of 26 detected cancers in total), and it was found to be stage II at the time of the positive Galleri test. However, there was also one false negative for an individual who was diagnosed with stage III PC who had a negative Galleri test [108]. While maintaining high specificity (99.5%) across cancer types and ages, the sensitivity of Galleri for stage I PC is 61.9%, for stage II PC 60.0%, for stage III PC 85.7%, and for stage IV PC 95.9% [110]. Overall, for early-stage PC, the sensitivity levels are low, leaving uncertainty about its clinical utility in HRIs undergoing PC surveillance.

A more recent SCED test, Avantect, utilizes epigenomic and genomic profiles of ctDNA associated specifically with PC with a goal of early PC detection. By using a combination of 5-hydroxymethylcytosine (5hmC) and whole-genome sequencing, Avantect has a sensitivity of 66.7% and a specificity of 96.9% in an initial study [111, 112]. However, prospective studies in HRIs are still needed.

Although many cancers detected on ctDNA tests are early stages, few are stage I and the overall performance of the test varies by cancer type; therefore, ctDNA tests must continue to be refined. With PC, where diagnosis at stage I is crucial, less than half of patients with a resectable PC have detectable ctDNA, and less than a third of those with stage I PC have detectable ctDNA [107]. Additionally, since circulating DNA mostly arises from leukocytes, some abnormal ctDNA results may arise from clonal hematopoiesis or other blood abnormalities, rather than a solid tumor [113, 114]. It also remains a question as to why some patients with cancer have a low ctDNA signal, which is one reason tests for early detection of cancer have started including aneuploidy detection in addition to mutations.

MCED may have more clinical utility in higher risk cohorts since these patients are typically at increased risks for several types of cancer, some of which have no effective screening method. More research is needed to determine how MCED tests can complement current surveillance protocols, and further examination of these tests is needed in longitudinal cohorts before regularly recommending them for routine use in PC surveillance.

### Cost-Effectiveness

There has been concern about the cost-effectiveness of PC surveillance, especially in those individuals who may have moderately, but not highly, elevated risks. The cost-effectiveness of PC surveillance has been recently reviewed in detail [115]. A recent comparison of no surveillance, endoscopic ultrasound, and MRI using a Markov model yielded some helpful results when considering different surveillance modalities. When analyzing those with a fivefold increased risk of PC, MRI is the most cost-effective strategy, while for those who have greater than a 20-fold relative risk, EUS is more cost-effective. Further analysis showed that if the cost of the MRI increased above $1600 USD, EUS then becomes more cost-effective [116]. Another study found that screening with EUS was cost-effective, especially when the lifetime risk of PC exceeds 10.8%, or if life expectancy after resection of a lesion was predicted to be at least 16 years [117]. Cost-effectiveness models are very sensitive to lifetime risk of PC, the probability of future PC diagnosis after a normal index imaging test, length of survival after resection, and the probability of a missed lesion [116]. Multiple studies have found that after age 76, surveillance is no longer cost effective; however, current guidelines do not specify an age at which PC surveillance should be stopped [116, 117]. Overall, it may be helpful to consider lifetime risk estimates rather than solely specific gene mutations in deciding which PC surveillance strategies are most cost-effective.

## Outcomes of Pancreatic Surveillance

Compared to screening strategies for other cancers such as breast and colorectal cancer, the outcomes data for PC surveillance is derived from small cohorts and is therefore far more limited. There have been only a handful of impactful studies conducted assessing the long-term outcomes of PC surveillance in HRIs. The data from these studies has been used to inform PC surveillance practices and is vital in understanding the key metrics of PC surveillance such as stage of diagnosis of surveillance-detected PCs, resectability rates, overall survival, and the rate of unnecessary surgical intervention. The outcomes of these studies and their findings for these key metrics are summarized below (Table 4).

**Table 4:** Studies assessing outcomes of pancreatic surveillance in high-risk individuals

Study	Total participants	Total number of participants diagnosed with PC	Total stage I, eighth AJCC	PC resection rate	5-year survival rate	Total number resections	Total number resection with high-risk pancreatic lesion or non-PC cancer	Total number resection for benign disease
Canto et al. 2019 (CAPS 1-4)	354	14 (4.0%)^a^	2 (14.3%)	10 (71.4%)	85%^b^	44	16 (4.5%) 6 NET	18 (5.1%)
Dbouk et al. 2024 (CAPS5)	1461	10 (0.68%)^c^	7 (70%)	8 (80%)	73.3%	16	3 (0.21%)	5 (0.34%)
Vasen et al. 2016 (*CDKN2A*)	178	13 (7.3%)	6 (42.8%)^d^	10 (76.9%)^e^	24%	12	0 (0.0%)	2 (1.1%)
Vasen et al. 2016 (all patients)	411	15 (3.6%)	7 (43.8%)^d^	12 (80.0%)^e^	Not calculated	31	5 (1.2%) 1 NET	14 (3.4%)
Overbeek et al. 2022	366	10 (2.7%)	4 (40%)	6 (60%)	Not calculated	17	4 (1.1%) 4 NET	7 (1.9%)
Klatte et al. 2022	347	31 (8.9%)^f^	16 (44.4%)	27 (75%)	44.1%	36	2 (0.58%) 1 ampullary cancer 1 NET	7 (2%)
Paiella et al. 2024	156	8 (5.1%)1 IPMN with invasive carcinoma	3 (37.5%)	6 (75%)	Not calculated	11	3 (1.9%)1 ampullary cancer1 NET	2 (1.3%)

[63, 64, 74, 118–120]

^a^Four PCs detected outside of study surveillance reported

^b^Three-year survival rate

^c^One PC detected outside of study surveillance reported

^d^One individual with second primary PC during study

^e^One PC diagnosis removed from resection rate analysis by Vasen et al. Resectable tumor identified in patient who opted out of surgery due to metastatic melanoma

^f^Five of the participants were diagnosed with a second primary PC during the study

### Rate and Stage of PC Diagnosis During Surveillance

Longitudinal studies of HRIs undergoing annual PC surveillance are critical to determine the rate of PC development and the stage of PC diagnosis in those undergoing surveillance. The United States-based Cancer of Pancreas Screening 5 (CAPS5) study reported on 1461 high-risk individuals enrolled, with 10 (0.68%) being diagnosed with PC during surveillance. Seven (70%) of the 10 individuals diagnosed with PC had stage I disease. Of note, one individual reported in the study had stage IV disease after dropping out of surveillance for 4 years. Combining the CAPS1-4 cohorts reported by Canto et al. in 2019 and two PC diagnoses identified after the last report of these cohorts with the CAPS5 cohort brings the total CAPS cohort to 1731 patients and 26 (1.5%) PC cases diagnosed, with 19 (73.1%) identified during surveillance and 11 (57.9%) having stage I disease [64, 120]. When compared to SEER data, the CAPS cohort was more likely to be diagnosed at a lower stage [121].

Within European studies, similar findings of higher rates of low-stage PC are observed. A 2016 study by Vasen et al. with data from Germany, Spain, and the Netherlands included 411 HRIs with *CDKN2A*, *BRCA1*, *BRCA2*, or *PALB2* PGVs or who met FPC criteria. 15 (3.6%) of the 411 individuals were diagnosed with PC during this study, with 76 (43.8%) being diagnosed with stage I disease [118]. In a 13-year prospective study by Overbeek et al. from the Netherlands, 366 individuals were followed with pancreatic surveillance with 10 (2.7%) individuals diagnosed with PC during this study, 4 (40%) being diagnosed at stage I [74]. Another study from the Netherlands by Klatte et al. in 2022 following 347 *CDKN2A* PGV carriers identified PC in 31 (8.9%) individuals, with 5 of these individuals having a second PC diagnosed on follow up surveillance. In this study, 16 (44.4%) of the 36 total PC diagnoses were stage I (using 8th AJCC) [63]. In a study from Italy by Paiella et al., 156 HRIs were followed with PC identified in 8 (5.1%) individuals, 3 (37.5%) being stage I. Of note, all late-stage PC diagnoses were identified on baseline surveillance, with only one stage I diagnosis being identified on baseline surveillance. On follow up surveillance, two stage I and one stage II were identified [119].

It is important to note that neoplasia can develop between surveillance exams, even without a previously observed high-risk lesion. Within a meta-analysis of 16 international studies and 2552 HRIs, Overbeek et al. identified 28 (1%) individuals who developed PC or high-grade dysplasia during surveillance. Of these 28 individuals, 13 (46%) had no lesions identified on their prior surveillance performed a median of 11 months prior to their PC diagnosis. At the time of diagnosis, 10 (77%) of the 13 had disease beyond the pancreas. For the remaining 15 (54%) individuals, a previous lesion was identified on surveillance, with 11 (73%) having disease beyond the pancreas at the time of PC diagnosis [122]. With nearly half these individuals having no previous lesion identified and the high rate of disease beyond the pancreas seen at the time of diagnosis, more sensitive diagnostic tools and risk-stratifying strategies are needed.

Among these studies, the rate of PC development during surveillance was low. Additionally, lower stage disease was identified more frequently in individuals undergoing surveillance compared to those not undergoing surveillance. However, not all PC diagnoses identified during surveillance are low-stage nor have a previous lesion observed on a prior surveillance exam, highlighting that improvements in early detection strategies and technologies are still direly needed.

### Resection Rate and 5-Year Survival Rates Among HRIs Undergoing Surveillance

Across all individuals with PC, approximately 80–85% of those presenting with a new PC diagnosis would be considered unresectable or metastatic at the time of diagnosis [123]. This high rate of unresectable disease contributes to the poor prognosis of PC and the 12.8% 5-year survival rate [1]. While long-term PC surveillance studies identify lower stage PC more often, it is important to ensure that they also show improvement in the PC resection rate and overall survival within their cohorts.

The CAPS5 study with 10 affected individuals saw 8 (80%) PC resections and a 5-year survival rate of 73.3% [64, 121]. Vasen et al. reported 13 affected *CDKN2A* PGV carriers with 10 resections being completed. One of the affected individuals has a secondary PC diagnosed and resected, so this resection rate was 71.4% (*n* = 10) for 14 PC diagnoses. Within this study, one of the 13 affected *CDKN2A* PGV carriers had resectable disease and opted to defer surgery, leading to the researchers reporting a 75% resection rate per affected individual (9 of 12 individuals), with 10 (76.9%) of the 13 resectable tumors being removed. The 5-year survival rate reported was 24% [118]. For all of the HRI individuals included in Vasen et al. 15 total PC diagnoses were reported with 12 (80.0%) having PC resection. However, 5-year survival was not reported for the collective HRI cohort [118]. Klatte et al. reported 31 individuals with a diagnosis of PC, with 5 of these individuals having a second primary PC identified on follow up surveillance. In total, 27 (75%%) of the 36 PC diagnoses were resected and a 5-year survival rate of 44.1% was reported for those completing resection [63]. Overbeek et al. and Paiella et al. only reported resection rates, with 6 (60%) of 10 and 6 (75%) of 8 PC diagnoses being resected, respectively [74, 119].

Overall, these studies reported higher rate of resectable PC than would be expected outside of surveillance, and those that did report 5-year survival rates were higher than the current 12.8% 5-year survival for all PC. Together, this data supports that the increased identification of early-stage PC on surveillance is associated with higher rates of resection and improved survival.

### Resection of Non-Neoplastic Pancreatic Lesions

One important risk of surveillance is the potential for a HRI to undergo surgical resection of a lesion that ultimately turns out to not be a high-risk pancreatic lesion. High-risk lesions that merit resection include PC, pancreatic intraepithelial neoplasia 3 (PanIN-3), or a lesion with high-grade dysplasia. However, it is possible for a HRI to undergo a pancreatic resection where none of these high-risk lesions are identified within the resection specimen.

Within the CAPS5 study, 5 (0.34%) total participants had a pancreatic resection without a high-risk lesion identified compared to 10 (0.68%) with a PC diagnosis [64]. Vasen et al. reported 14 (3.4%) individuals with benign pancreatic resections compared to 15 (3.6%) among their entire studied cohort with a PC diagnosis [118]. Of note, within their *CDKN2A* cohort, 2 (1.1%) individuals had benign resected disease compared to 13 (7.3%) with a PC diagnosis [118]. The two studies from the Netherlands completed by Overbeek et al. and Klatte et al. reported 7 (1.9%) individuals with benign disease on resection compared to 10 (2.7%) with a PC diagnosed and 7 (2%) individuals with benign disease compared to 31 (8.9%) with a PC diagnosed, respectively [63, 74]. Finally, Paiella et al. reported 2 (1.3%) individuals with benign disease compared to 8 (5.1%) with a diagnosis of PC [119].

Across these studies, the total number of individuals completing resection for benign disease was typically lower than the total number of individuals diagnosed with PC. A recent large meta-analysis by Piaella et al. (2024) also assessed the rate of benign resections, finding a pooled prevalence of a pancreatic resection with benign findings of 2.1% across 23 articles with 5027 patients [124]. Importantly, this rate has decreased over time with more recent data showing a 1% rate of pancreatic resection with benign findings, which is likely due to improved modalities for PC diagnosis and improved patient selection for surgery. Nonetheless, the potential for a pancreatic resection for benign disease must be considered when discussing pancreatic surveillance with HRIs so they may weigh the benefits of identifying PC early to the potential harms of undergoing a resection for benign disease.

### Surgical Outcomes and Cancer Worry

Pancreatic resection outcomes have improved over time; however, there is still substantial surgical morbidity, which HRIs should be educated on. Using prospective CAPS data from 1998 to 2014, outcomes from pancreatic resections following a distal pancreatectomy, Whipple, or total pancreatectomy were studied for 48 individuals. No perioperative deaths were seen, 17 (35%) of the 48 reported a post-operative complication, and 6 (20%) of the 30 who completed partial pancreatic resection developed post-operative diabetes mellitus [125]. The median length of hospital stay after resection was 7 days, with those completing total pancreatectomy having a median of 11.5 days, and a total of three (6.2%) readmissions for post-operative complications were seen [125].

As for cancer worry, in a 2016 study, 166 individuals undergoing annual PC surveillance were asked to complete questionnaires assessing factors impacting cancer worry from time of discussing surveillance to the second annual surveillance. This study indicated that detection of cystic lesions, increasing the interval of surveillance, or undergoing pancreatic surgery did not significantly increase cancer worry, with the only factor significantly increasing perceived risk being a family history of PC under the age of 50 [126]. Similar findings were reported in a 2023 study on 100 HRIs completing EUS surveillance, where a family history of PC and lower self-reported mental health score were predictors for an increased perceived risk of PC [127]. This study also identified a 53.4% decrease in distress levels following surveillance which were sustained after completing a surveillance exam [127].

Together, this data allows providers the opportunity to inform their patients about possible post-operative risks associated with pancreatic resections. Furthermore, these data also support that completing PC surveillance does not have a negative psychosocial impact on HRIs and in fact may provide some psychological benefit.

## Conclusion

Pancreatic surveillance is being increasingly performed in HRIs, providing hope of PC early detection to this high-risk cohort. However, there remains debate about which HRIs should be eligible for surveillance, when surveillance should start and stop, and how surveillance should be performed. Further study of surveillance outcomes will be critical to answer these questions and optimize how PC surveillance is performed.

## Data Availability

No datasets were generated or analysed during the current study.
